# Identifying of biomarkers associated with gastric cancer based on 11 topological analysis methods of CytoHubba

**DOI:** 10.1038/s41598-020-79235-9

**Published:** 2021-01-14

**Authors:** Hua Ma, Zhihui He, Jing Chen, Xu Zhang, Pingping Song

**Affiliations:** 1 School of Mathematics and Statistics, Southwest University, Chongqing, 400715 China; 2 Department of Pediatric Respiration, Chongqing Ninth People’s Hospital, Chongqing, 400700 China; 3 School of Science, Southwest University of Science and Technology, Sichuan, 621000 China

**Keywords:** Computational biology and bioinformatics, Mathematics and computing

## Abstract

Gastric cancer (GC) is one of the most common types of malignancy. Its potential molecular mechanism has not been clarified. In this study, we aimed to explore potential biomarkers and prognosis-related hub genes associated with GC. The gene chip dataset GSE79973 was downloaded from the GEO datasets and limma package was used to identify the differentially expressed genes (DEGs). A total of 1269 up-regulated and 330 down-regulated genes were identified. The protein-protein interactions (PPI) network of DEGs was constructed by STRING V11 database, and 11 hub genes were selected through intersection of 11 topological analysis methods of CytoHubba in Cytoscape plug-in. All the 11 selected hub genes were found in the module with the highest score from PPI network of all DEGs by the molecular complex detection (MCODE) clustering algorithm. In order to explore the role of the 11 hub genes, we performed GO function and KEGG pathway analysis for them and found that the genes were enriched in a variety of functions and pathways among which cellular senescence, cell cycle, viral carcinogenesis and p53 signaling pathway were the most associated with GC. Kaplan-Meier analysis revealed that 10 out of the 11 hub genes were related to the overall survival of GC patients. Further, seven of the 11 selected hub genes were verified significantly correlated with GC by uni- or multivariable Cox model and LASSO regression analysis including C3, CDK1, FN1, CCNB1, CDC20, BUB1B and MAD2L1. C3, CDK1, FN1, CCNB1, CDC20, BUB1B and MAD2L1 may serve as potential prognostic biomarkers and therapeutic targets for GC.

## Introduction

Gastric cancer (GC), a common heterogeneous disease, is one of the most deadly malignancies worldwide, especially in East Asia^1^. Previously, many patients missed the optimal diagnosis and treatment time, which leads to tumor cell metastasis and progression to advanced cancer. Currently, there is a lack of effective biomarkers for early diagnosis. Comprehensive treatment and cancer surveillance have been identified as one of the major obstacles to improve the prognosis of gastric cancer^2^. Therefore, a deeper understanding of the mechanisms involved in GC progression and identification of potential biomarkers and targets for the diagnosis, prognosis and therapy of GC are urgently needed.

Bioinformatics analysis methods, are powerful tools for identifying potential biomarkers related to diagnosis and treatment, including the analysis of gene interaction networks, gene annotation and microarray expression profiles^3^. For example, Hao et al. explored 10 genes (COL1A1, COL3A1, COL1A2, COL5A2, FN1, THBS1, COL5A1, SPARC, COL18A1 and COL11A1) as potential biomarkers and therapeutic targets for GC, through analysis data from the Gene Expression Omnibus (GEO) database^4^. Furthermore, Zhu et al. found that CDK1 overexpression was a prognostic factor for hepatocellular carcinoma (HCC), which makes it a potential therapeutic target and biomarker for HCC diagnosis, through analysis data from GEO and the Cancer Genome Atlas (TCGA)^5^. Liao et al. identified two genes (SERPINE1 and SPARC) as potential biomarkers and therapeutic targets for GC^6^. However, the methods of hub gene selection in the above literatures was single and the potential molecular mechanism of gastric cancer was still unclear, which needs further exploration.

In this study, DEGs between human GC tissues and normal tissues were identified using Limma based on GEO datasets. And a directed acyclic graph was constructed with Bingo plug-in to view the overall enrichment of DEGs. Next we constructed a PPI network of DEGs based on the STRING V11 database and visualized it using Cytoscape software. And then 11 topological analysis methods were adopted to select the hub genes. Important module in the network related to the hub genes were abstracted by MCODE. In addition, in order to explore the role of the 11 selected hub genes in the pathogenesis of GC, we performed GO function and KEGG pathway enrichment analysis for the genes. Finally, Kaplan-Meier analysis was performed to evaluate the prognostic value of these hub genes. And Cox model and LASSO regression analysis were used to verify these hub genes further.

## Results

### Data preprocessing

**Figure 1:**
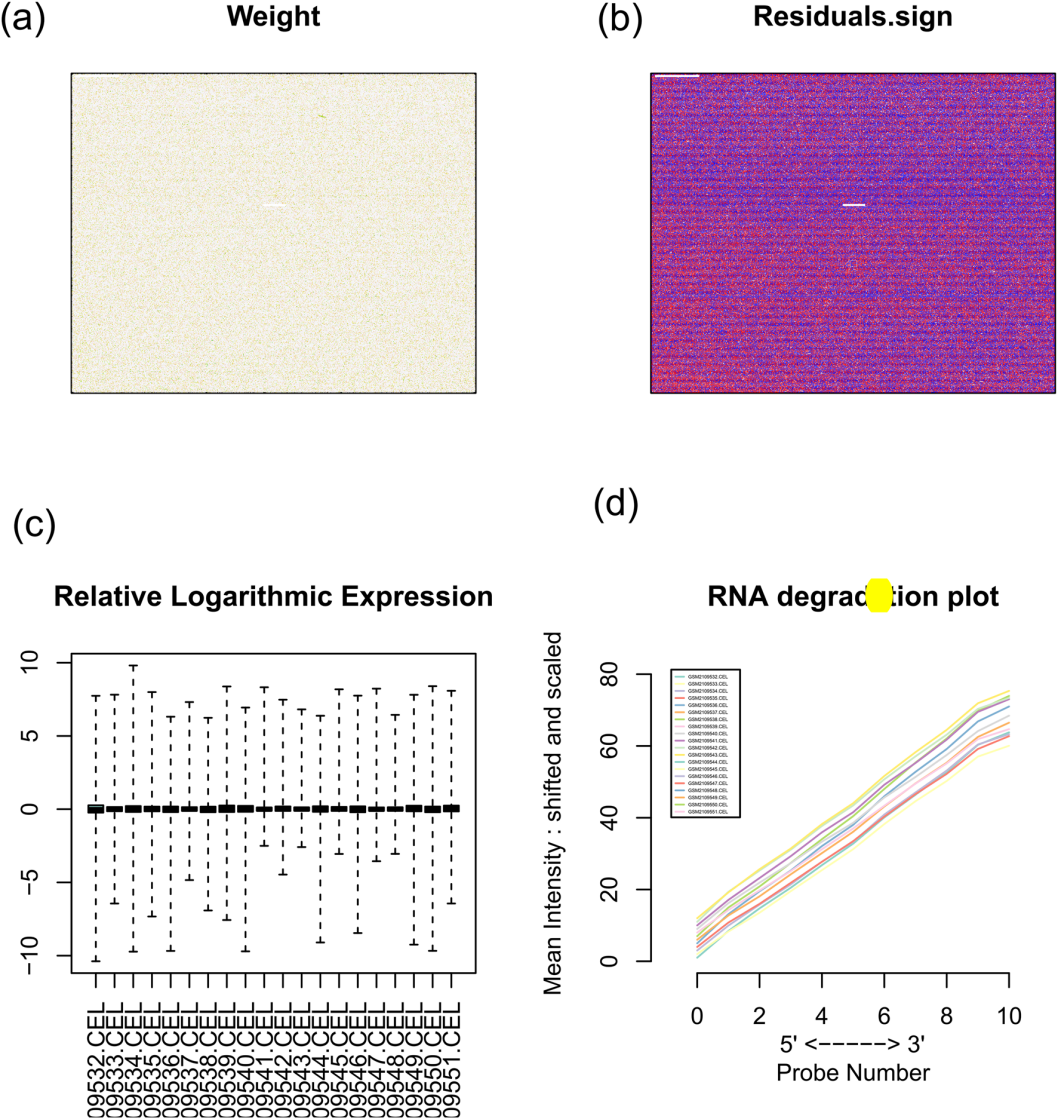
Maps to check the quality of the data. (**a**) is a weight map, (**b**) is a residual symbol map, (**c**) is a relative logarithmic expression and (**d**) is an RNA degradation map. [The figures were created by R version 3.5.1 (https://www.r-project.org/)].

It can be seen from the weight and residual symbol maps (Fig. 1a–b) that all the points were evenly distributed. In the relative logarithmic expression graph (Fig. 1c), all the samples were near the zero point without outliers. For the RNA degradation diagram (Fig. 1d), generally we need 5′-terminal lower than 3′-terminal^7^. Our results showed that the data was in good quality and suitable for downstream analysis.

### Differentially expressed genes and enrichment analysis

**Figure 2:**
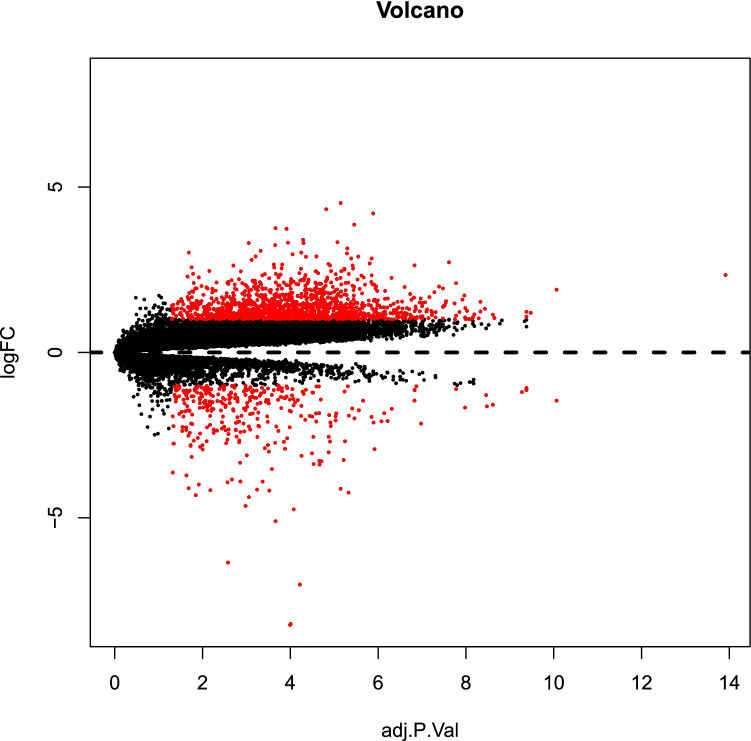
Volcanic map of DEGs, red color represents DEGs in GC including up-regulated and down-regulated genes. [The figure was created by Limma (http://www.bioconductor.org/packages/release/bioc/html/limma.html)].

**Figure 3:**
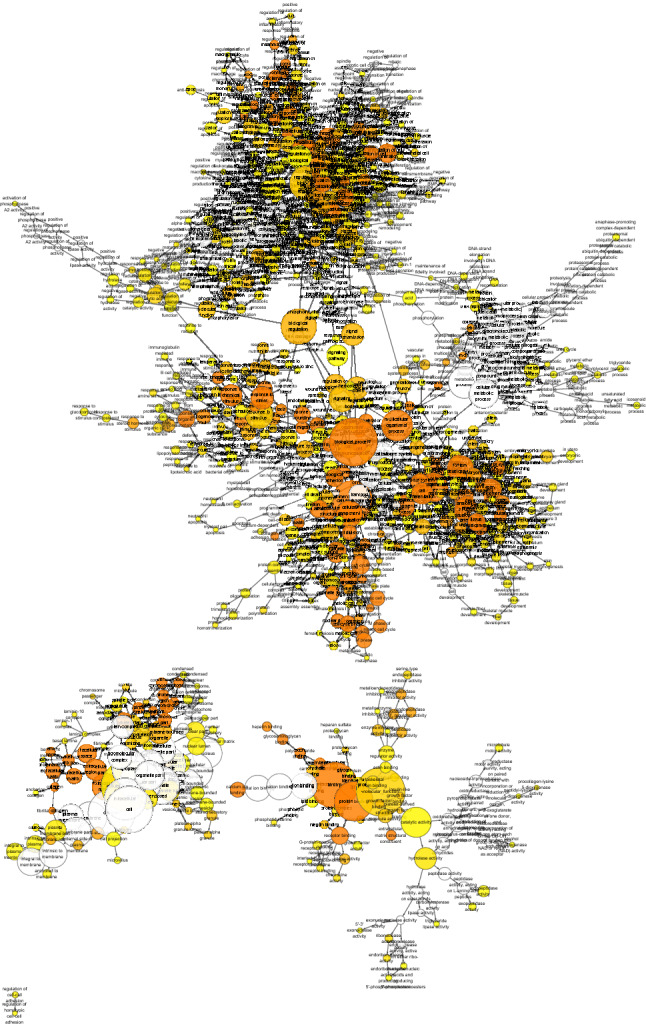
Enrichment analysis of DEGs. The deeper color the higher degree of enrichment. [The figure was created by Bingo plug-in in Cytoscape (https://cytoscape.org/)].

Though Limma method, a total of 1599 DEGs were identified in the dataset GSE79973, of which 1269 were up-regulated and 330 were down-regulated (Fig. 2). Cytoscape’s plug-in Bingo generated a directed acyclic graph (Fig. 3), in which branches represented inclusion relationships. The range of functions defined by the arrow direction from top to bottom was getting smaller and smaller, the deeper color the higher degree of enrichment. The graph was divided into three parts, representing BP, MF and CC. These genes were thought to be involved in the regulation of RNA metabolic processes, positive and negative regulation of phosphorous metabolic processes in the adaptive immune response based on somatic cells, extracellular matrix tissue and etc.

### PPI network and GC-associated clustering module construction

**Figure 4:**
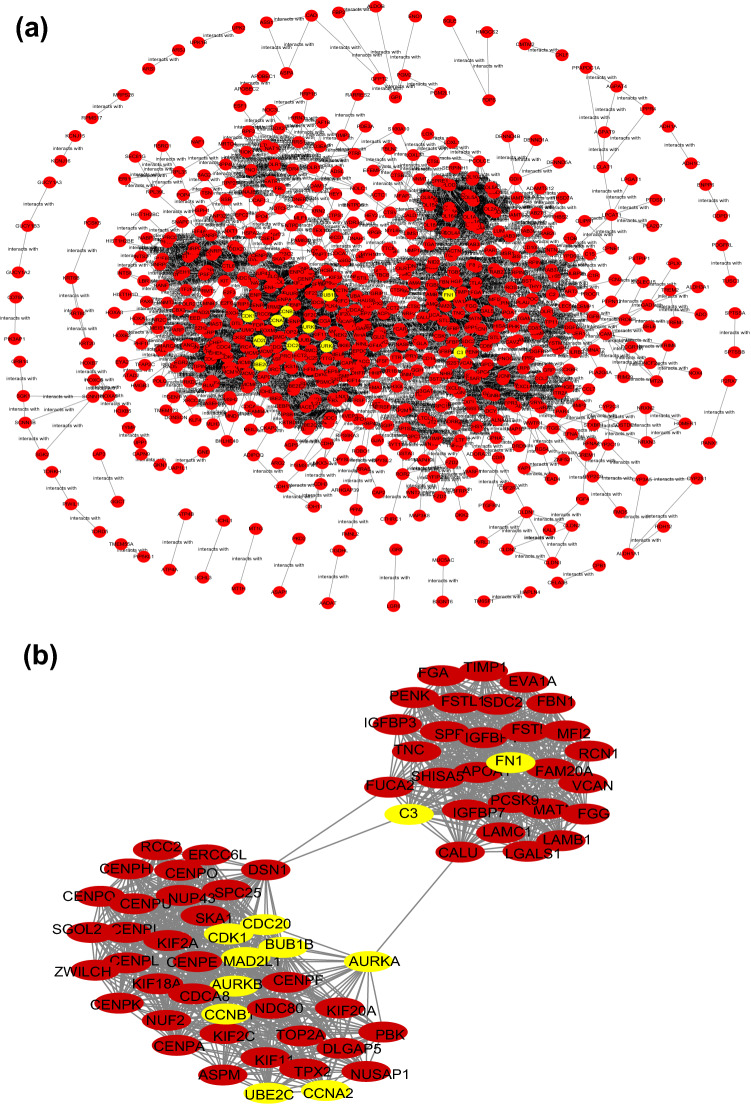
PPI network and highest score clustering module. (**a**) PPI network, yellow color indicates the hub genes, red nodes represent DEGs. (**b**) The highest score clustering module generated by MCODE, yellow color indicates the hub genes, red nodes represent DEGs. [The figures were created by STRING V11.0 (https://string-db.org/) and MCODE plug-in in Cytoscape (https://cytoscape.org/)].

Based on 1599 DEGs, a PPI network was constructed where 1376 genes formed the network with 14,394 edges (Fig. 4a). And hub genes were identified by 11 topological analysis methods, where the top 20 genes were selected for each method (Supplementary file 1), among which C3, CDK1, AURKA, CDC20, CCNA2, AURKB, CCNB1, BUB1B, MAD2L1, UBE2C and FN1 were found in the intersection of at least five methods and were selected as GC related hub genes. We also obtained the clustering module with the highest score from PPI network of all DEGs (Fig. 4b) by MCODE algorithm. It was found that all the 11 hub genes were contained in this module.

### GO and KEGG functional enrichment analysis for the selected hub genes

**Figure 5:**
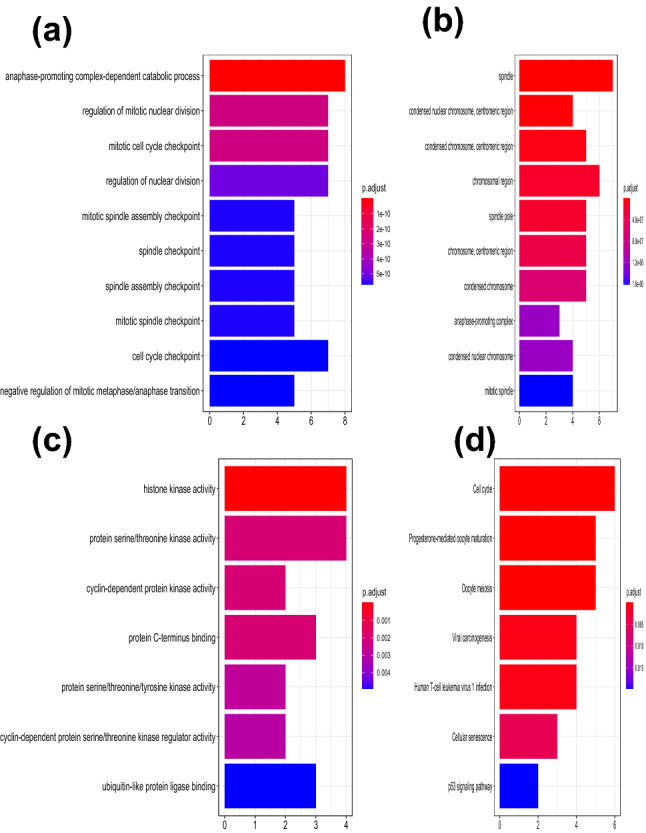
GO and KEGG functional enrichment analysis. (**a**) Top 10 terms in BP category. (**b**) Top 10 terms in CC category. (**c**) All terms in MF category. (**d**) All terms in KEGG pathway analysis. (The figures were created by R version 3.5.1 based on the KEGG pathway database www.kegg.jp/kegg/kegg1.html).

In order to explore the role of the 11 selected hub genes, we performed GO function and KEGG pathway analysis for them. The results of GO function enrichment indicated GO terms for 187 biological processes (BP), 29 cell components (CC) and 7 molecular functions (MF), see Supplementary file 2. Figure 5 showed the top 10 terms for BP, CC and all the terms for MF and KEGG. Among these top terms, we found 6 terms associated with GC and some similar cancers. These terms were mitotic spindle checkpoint, anaphase-promoting complex, cell cycle, viral carcinogenesis, cellular senescence and p53 signaling pathway. Kim et al. found that frequent mutations of human Mad2, but not Bub1, in gastric cancers cause defective mitotic spindle checkpoint^8^. Zhu et al. found that the miR-383 inhibited the cell cycle progression of gastric cancer cells via targeting cyclin E2^9^. Uozaki et al. studied that gastric cancer and viral carcinogenesis through epigenetic mechanism^10^. Ji et al. found that microRNA miR-34 was a direct target of p53, which played an anti-cancer role in the downstream of p53 pathway^11^. By modulating the cellular senescence through E2F/miR-106b-5p/p21 axis, Dong et al. found a novel mechanism by which BRD4 regulated cancer cell proliferation and provided new insights into using BET inhibitors as potential anticancer drugs^12^. Dai et al. found that activation of anaphase-promoting complex by p53 induced a state of dormancy in cancer cells against chemotherapeutic stress^13^.

### Survival analysis

**Figure 6:**
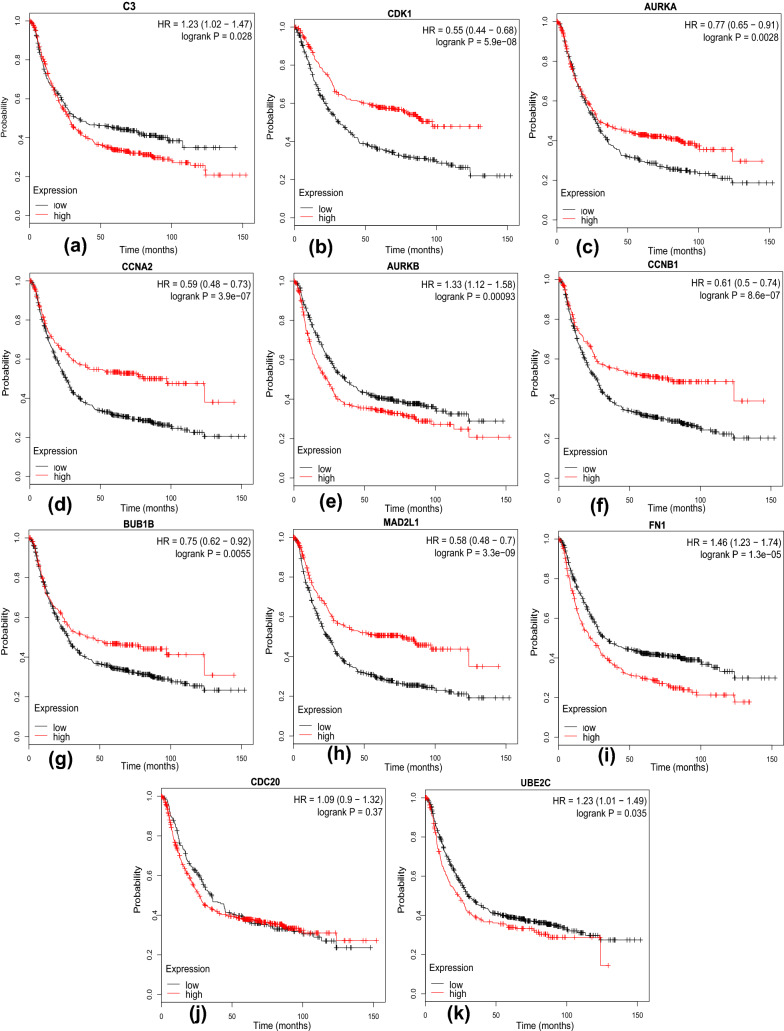
Survival plot of the significant genes by Kaplan Meier test. The Kaplan–Meier test p-value < 0.05: (**a**) C3, (**b**) CDK1, (**c**) AURKA, (**d**) CCNA2, (**e**) AURKB, (**f**) CCNB1, (**g**) BUB1B, (**h**) MAD2L1, (**i**) FN1 and (**k**) UBE2C. The test p-value > 0.05: (**j**) CDC20. [The figures were created by Kaplan–Meier (https://kmplot.com/analysis/index.php?p=service&cancer=gastric)].

Using the Kaplan–Meier plotter database, the prognostic value of the 11 hub genes were evaluated in GC patients. All the 11 hub genes were up-regulated ($log_{2} fold change (FC)>1$, Table 1). It was found that 10 out of the 11 hub genes including C3, CDK1, AURKA, CCNA2, AURKB, CCNB1, BUB1B, MAD2L1, FN1 and UBE2C (p-value < 0.05) had significant difference of overall survival between high and low expression. The results showed that the survival rate of high expression C3, AURKB, FN1 and UBE2C groups were significantly lower than that of low expression groups, and the survival rate of low expression CDK1, AURKA, CCNA2, CCNB1, BUB1B, MAD2L1 groups were significantly lower than that of high expression (Fig. 6).

**Table 1:** Regulation of 11 hub genes.

Gene symbol	Log FC	p value	Adj p val
FN1	2.182008173	5.82E-−06	4.96E−05
UBE2C	2.1341329	0.000173126	0.000693925
CCNB1	1.736845192	0.000104015	0.000459134
MAD2L1	1.635046726	2.17E−05	0.000133896
CCNA2	1.582022494	4.42E−05	0.000231949
C3	1.561270443	0.006073294	0.013605247
CDK1	1.321178004	0.002097257	0.005543793
BUB1B	1.318731789	0.002695901	0.006858173
CDC20	1.301949207	0.005086582	0.011692519
AURKB	1.240762245	4.32E−05	0.000227121
AURKA	1.023628153	0.002870315	0.007224631

### Verification based on another dataset

Based on dataset GSE19826, Limma identified 333 DEGs. These DEGs were imported into the STRING V11 database to obtain a TSV file of protein interactions. And hub genes were identified by 11 topological analysis methods, where the top 20 genes were selected for each method (Supplementary file 3), among which FN1, CDK1, MMP9, CCNB1, AURKA, UBE2C, AURKB, CCNA2, FOXM1, ITGB1, EZH2, RRM2, THBS1, CXCL8, CDC20, COL1A1, BUB1B, MAD2L1, HIF1A and CDH2 were found in the intersection of at least five methods, including 10 out of the 11 hub genes in the above conclusion, except C3. Important clustering module related to the 11 hub genes were abstracted by CytoHubba which had 133 nodes and 1077 edges (Supplementary Fig. 1a). We obtained two clustering modules with the highest score from PPI network of all DEGs (Supplementary Fig. 1b,c) by MCODE algorithm. It was found that all the 11 hub genes were contained in this module.

### Cox analysis and LASSO regression analysis

**Figure 7:**
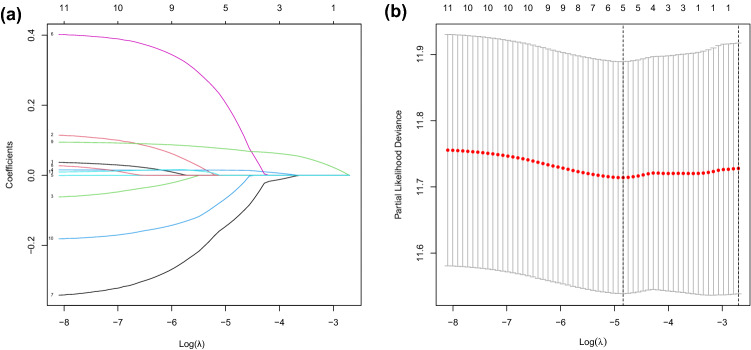
LASSO regression model analysis. (**a**) is the path change chart of regression coefficient and (**b**) is the change curve of penalty term. [The figure was created by R version 3.5.1 (https://www.r-project.org/)].

The analysis results of correlation between DEGs expression and overall survival (OS) as well as other clinical features investigated by Cox analysis were shown in Table 2. The results suggested that six clinical features including age, stage, grade, T, M and N and six hub genes including C3, CDK1, FN1, CCNB1, CDC20 and BUB1B were revealed significantly correlated with OS (p-value < 0.05) by univariate or multivariate Cox analysis. The LASSO method established regression model and continued to screen the 11 hub genes. By setting different $\lambda$, the path change graph of the regression coefficient was obtained (Fig. 7a). The trend of each curve in the figure represented the change of the regression coefficient path. It could be seen that the regression coefficients were mostly compressed to zero, which showed that the model had a good advantage in dimensionality reduction and variable selection. Each point in Fig. 7b corresponded to a penalty value, and the position of the vertical dashed line represented the number of genes selected under the optimal model. It could be seen from the figure that there were five genes under the optimal model, including C3, CCNB1, CDC20, FN1 and MAD2L1. Therefore, seven of the 11 selected hub genes including C3, CDK1, FN1, CCNB1, CDC20, BUB1B and MAD2L1 were verified through Cox analysis or LASSO regression analysis which could be taken as independent prognostic biomarkers for GC.

**Table 2:** Univariate and multivariate Cox analysis.

Variables	Univariate Cox				Multivariate Cox				
	HR	HR.95L	HR.95H	p value	Variables	HR	HR.95L	HR.95H	p value
Age	1.022212351	1.005811513	1.038880621	0.007764558	Age	1.03433333	1.016841941	1.052125601	0.000104766
Grade	1.420247616	1.023095183	1.971569532	0.036046698	Grade	1.567172974	1.103167545	2.226344622	0.012138576
Stage	1.653117193	1.348649804	2.026320283	1.30E-06	Stage	1.353901129	1.024986088	1.788364047	0.032862236
T	1.39801979	1.137483947	1.718230255	0.001451642	M	2.195910547	1.337728548	3.60463499	0.001866895
M	2.372585415	1.502749378	3.745908422	0.000208927	C3	1.001531756	1.00010612	1.002959424	0.035207018
N	1.387710481	1.195331802	1.611050903	1.68E-05	CDK1	0.958783737	0.928669867	0.989874107	0.009736787
C3	1.161967017	1.048765553	1.2873872	0.004099372	BUB1B	8.568665988	1.604562951	45.75827753	0.011965015
CDK1	1.129353067	1.012197799	1.260068291	0.029485595	FN1	1.100769547	1.00269362	1.20843852	0.043751763
FN1	1.125876038	1.008258757	1.25721383	0.035198627	CCNB1	1.478932638	1.056967554	2.069355618	0.022416923
BUB1B	1.122389719	1.00533721	1.25307078	0.03990889	CDC20	0.689337825	0.509422258	0.932795201	0.015918054

## Discussion

The study of molecular genetics and signal transduction pathways are helpful for further understanding of the pathogenesis and early diagnosis of GC. Therefore, recognition of DEGs for GC based on transcriptome microarray datasets may contribute to early diagnosis and develop effective therapies.

In our study, a total of 1599 DEGs were identified in the dataset GSE79973, of which 1269 were up-regulated and 330 were down-regulated. Based on these DEGs, a PPI network was constructed where 1376 genes formed the network with 14,394 edges. And 11 hub genes were selected through intersection of 11 topological analysis methods. Clustering module related to these 11 genes were obtained by MCODE. In order to explore the role of the 11 selected hub genes in the pathogenesis of GC, we performed GO function and KEGG pathway enrichment analysis for them and found that the genes were enriched in a variety of functions and pathways. Kaplan–Meier analysis revealed that 10 out of the 11 hub genes were related to the overall survival of GC patients. Cox analysis and LASSO regression analysis showed that seven of the 11 selected hub genes were significantly correlated with GC.

The overall aim of this study was to identify the hub genes which may serve as potential biomarkers for GC diagnosis and therapy, and to further explore the potential mechanisms of GC by integrated profiling analysis. In our study, seven of the 11 selected hub genes including C3, CDK1, CCNB1, BUB1B, MAD2L1, FN1 and CDC20 were considered to be the most likely independent prognostic biomarkers associated with GC. And five of them were newly found including C3, CDC20, CCNB1, BUB1B and MAD2L1 compared with previously published results using the same dataset. Some relevant literatures suggested from biological point of view that most of these found hub genes played important roles on GC. Kitano et al. showed that the synthesis and secretion of C3 by all the tested GC derived cell lines in response to TNF, suggested that C3 may be secreted in the gastric wall as part of its normal physiology, or as a result of tumour pathology and thereby participate in local immune or inflammatory responses^14^. Lee et al. found that the high expression of CDK1 in GC patients may imply a strong biological ability of tumor invasion and CDK1 was the target gene of mir-490-5p. Down-regulation of mir-490-5p and up-regulation of CDK1 can promote the proliferation ability of GC cells and the transformation of *G*1/*S* phase^15^. A study by Kidokoro et al. found that CDC20 was often up-regulated in many types of tumors and significantly inhibited by ectopic introduction of p53. Additionally, treatment of cancer cells with siRNA against CDC20 can induce *G*2/*M* arrest and inhibit cell growth^16^. CCNB1 knockdown by RNA interference was found to significantly inhibited proliferation, migration and invasion of HCC cells^17^. Hudler et al. found that the expression of BUB1B in GC tissues was significantly higher than that in adjacent normal tissues, nearly $(8.875 \pm 1.08)$ times^18^. Frio et al. found that at least one BUB1B mutation can result in autosomal recessively inherited susceptibility to gastrointestinal cancer, as do mutations in MUTYH and the mismatch-repair genes^19^. FN1 was an significant regulatory factor promoting the development and formation of various cancer cells, such as laryngeal, skin squamous carcinoma^20,21^ and brain glioblastoma^22^. Zhang et al. found that miR-200c can inhibit the migration, proliferation and invasion of GC cells in vitro by directly combining with FN1, which indicated that mir-200c and FN1 may be potential biomarkers or therapeutic methods for GC^23^. A study by Wang et al. confirmed the prognostic value of two key mitotic checkpoint genes MAD2L1 and BUB1, which have been included in multiple gene expression signatures for breast cancer prognosis. And they also found that these genes were biologically relevant to breast cancer progression, as suppression of their expression was associated with reduced tumor cell growth, migration and invasion^24^.

This study provides important clues for exploring potential biomarkers and targets for the diagnosis, prognosis and treatment of GC. In future work, if condition permits, we hope to conduct some experiments to verify the important relation between these hub genes and GC from biological point of view.

## Conclusion

Through 11 topological analysis methods, we identified 11 hub genes for GC. We validated these hub genes through functional enrichment analysis, the clustering module with the highest score, relevant literatures, Kaplan–Meier analysis, Cox analysis and LASSO regression analysis. The results suggested that seven of the 11 selected hub genes including C3, CDK1, CCNB1, BUB1B, MAD2L1, FN1 and CDC20 may serve as potential prognostic biomarkers and therapeutic targets for GC. These results may provide a theoretical direction for future research with regards to the molecular mechanisms of the progression of GC.

## Materials and methods

### Dataset

We collected the set of gene expression profiles of GC from the Gene Expression Omnibus database (https://www.ncbi.nlm.nih.gov/geo/query/acc.cgi?acc=GSE79973). This dataset includes 10 GC samples and 10 normal gastric samples. The platform was GPL570 (Affymetrix Human Genome U133 Plus 2.0). This dataset was used by previous studies^4,6,25–27^, where the authors mainly clarified the emerging role of long non-coding RNA (lncRNA) in cancer development, explored novel lncRNA candidates, identified key candidate genes and circRNA, and explored the molecular mechanism of GC through comprehensive analysis of mRNA and miRNA expression profiles. Here we aimed to identify potential prognostic biomarkers of GC based on 11 topological analysis methods and used the MCODE method and survival analysis to verify these hub genes.

### Data preprocessing

The fitPLM function in the affyPLM package was adopted to perform regression calculation on the dataset. Then, weight map, residual symbol map, relative logarithmic expression map and RNA degradation map were used to check whether the data quality was suitable for downstream analysis. Finally, the normal samples and gastric cancer samples were processed by RMA method and the missing values were added by nearest neighbor method (KNN, k-Nearest Neighbor).

### Identification of DEGs and enrichment analysis

The lmFit and eBayes functions in the limma package were used to identify the DEGs between GC and control samples. The threshold values $log_{2} fold change (FC)>1$ and p-value < 0.05 indicated up-regulated DEGs, while $log_{2} (FC)<-1$ and p-value < 0.05 indicated down-regulated DEGs. we also used the Cytoscape plug-in Bingo to examine the enrichment of all DEGs in the biological processes of Biological Process (BP), Molecular Function (MF) and Cellular Component (CC)^28^.

### PPI network and GC-associated clustering module construction

We constructed a PPI network of DEGs using the STRING V11 database^29^. Based on the genes in the network, we searched for hub genes through 11 topological analysis methods^30^. The selected hub genes were verified by MCODE method.

### GO function and KEGG pathway enrichment analysis

To identify the potential functions of the selected hub genes, we used the clusterProfiler package in R to perform GO functions and KEGG pathway analysis of these genes^31^. ClusterProfiler is an R package of Bioconductor which can perform statistical analysis and visualization of functional clustering of gene sets or gene clusters.

### Survival analysis

The Kaplan–Meier plotter database (http://kmplot.com), an online tool to evaluate the prognostic values of genes in breast, ovarian, lung and gastric cancer patients, was applied to analyze the associations between the identified hub genes and overall survival^32^. The hazard ratio (HR) and its 0.95 confidence intervals were calculated. p < 0.05 was used to indicate a statistically significant difference^33^.

### Verification based on another dataset

In order to validate the efficiency of our method, another mRNA expression dataset (GSE19826) was downloaded from the GEO database (https://www.ncbi.nlm.nih.gov/geo/), the mRNA profifiles were based on the GPL570 platform (Affymetrix Human Genome U113 Plus 2.0 Array). The GSE19826 dataset included 15 paracancerous tissues and 12 tumor tissues. The same method was carried out on this dataset. Firstly, the Limma method was used to identify DEGs between GC tissues and paracancerous tissues. Secondly, a PPI network of DEGs was constructed based on the STRING V11 database. Based on the genes in the network, we searched for hub genes through 11 topological analysis methods in the CytoHubba. Then, the clustering module related to these hub genes were constructed by CytoHubba and the selected hub genes were verified by MCODE method.

### Cox analysis and LASSO regression analysis

The TCGA data with corresponding clinical features of GC was downloaded from TCGA database (https://tcgadata.nci.nih.gov/tcga/) which contained 375 tumor tissue samples and 32 paracancerous tissue samples. Cox proportional hazard models of univariate and multivariate were used to calculate 95% confidence interval (CI) and hazard ratio (HR) for the DEGs and clinical features where survival package was used for statistical analysis. Univariate Cox analysis model was used to compare the relationship between clinical features and survival rates. Multivariate Cox analysis model was used to evaluate how the genes expression and the clinical factors affect overall survival (OS). p < 0.05 was set as the threshold. LASSO regression analysis was applied to confirm the selected hub genes further. When solving the regression model, LASSO fitted the model that contained $\lambda$ prediction variables by constraining or punishing the coefficients and compressing the coefficient estimation value to zero direction, so that the model had a better interpretation effect. The screening of related variables could be done through the cv.glmnet and glmne function in R language.

### Consent for publication

All the authors have consented for the publication.

## Supplementary Information

Supplementary Information 1.

Supplementary Information 2.

Supplementary Information 3.

## Data Availability

The datasets used and analyzed during the current study are available online.

## Abbreviations

GC: Gastric cancer
DEGs: Differentially expressed genes
GEO: Gene expression omnibus
FC: Fold change
GO: Gene ontology
KEGG: Kyoto encyclopedia of genes and genomes
PPI: Protein–protein interactions
STRING: Search tool for the Retrieval of Interacting Genes
MCODE: The molecular complex detection
BP: Biological process
MF: Molecular function
CC: Cellular component
