# Supplementary material for: Identifying of biomarkers associated with gastric cancer based on 11 topological analysis methods of CytoHubba
Source: Sci Rep. 2021 Jan 14;11:1331. doi: 10.1038/s41598-020-79235-9 (PMC7809423; doi:10.1038/s41598-020-79235-9)
Supplement: Supplementary file 3 — Supplementary Information 3. [file 41598_2020_79235_MOESM3_ESM.pdf]

Identifying of biomarkers associated with gastric cancer based on 11 topological analysis methods of CytoHubba

Hua Ma[1], **Zhihui He**[2], **Jing Chen**[3], **and Xu Zhang**[1,*]

[1]School of mathematics and statistics, Southwest University,Chongqing,400715,China
[2]Department of Pediatric Respiration, Chongqing Ninth People's Hospital,Chongqing,400700,China
[3]School of Science, SouthWest University of Science and Technology,Sichuan,621000,China
[*]zhangxu1107@163.com

Supplementary table1. Top 20 in network ranked by Degree, EPC , MCC ,EcCentricity ,DMNC ,Closeness methods

Degree method		EPC method		MCC method		EcCentricity method		DMNC method		Closeness method	
Name	Score	Name	Score	Name	Score	Name	Score	Name	Score	Name	Score
FN1	72	FN1	63.735	CDK1	2.04E+20	CCNB1	0.248054475	TRIP13	1.202685709	FN1	152.8333333
CDK1	48	AURKB	61.779	BUB1B	2.04E+20	MMP9	0.248054475	RAD51AP1	1.184667946	MMP9	138.3333333
MMP9	46	CDK1	61.613	CDC20	2.04E+20	TIMP2	0.248054475	C3	1.163689242	ITGB1	130.1166667
CCNB1	40	CCNA2	60.582	CCNA2	2.04E+20	FN1	0.248054475	SMC2	1.130457567	AURKA	129.7833333
COL1A1	39	CCNB1	60.331	AURKB	2.04E+20	THBS1	0.248054475	ANLN	1.121605774	CDK1	129.3166667
AURKA	39	AURKA	59.684	CCNB1	2.04E+20	CXCL8	0.248054475	DLGAP5	1.109808627	AURKB	129.1166667
ITGB1	39	CDC20	59.367	CCNB2	2.04E+20	IFNL2	0.19844358	KPNA2	1.1052429	CCNB1	128.75
AURKB	38	CCNB2	58.854	MAD2L1	2.04E+20	ANPEP	0.19844358	CKS1B	1.087686196	CDH2	127.6166667
CCNA2	36	BUB1B	58.842	MCM2	2.04E+20	TGFBI	0.19844358	TYMS	1.061876693	CXCL8	127.0833333
BUB1B	35	UBE2C	58.782	UBE2C	2.04E+20	RNASE1	0.19844358	MCM2	1.06122327	THBS1	125.3333333
CDC20	34	MAD2L1	57.937	AURKA	2.04E+20	IGF2BP3	0.19844358	MAD2L1	1.038780451	COL1A1	124.45
TOP2A	34	MMP9	57.925	PTTG1	2.04E+20	MGP	0.19844358	CCNB2	1.014528763	FOXM1	123.9333333
EZH2	34	TOP2A	57.865	DLGAP5	2.04E+20	WASF3	0.19844358	PTTG1	1.007956105	CCNA2	123.9
UBE2C	33	FOXM1	57.45	TOP2A	2.04E+20	RCN1	0.19844358	CDC25B	0.996136855	EZH2	122.4333333
MAD2L1	33	EZH2	57.435	RRM2	2.04E+20	CDK1	0.19844358	RRM2	0.943224979	HIF1A	122.2833333
FOXM1	33	MCM2	57.404	TYMS	2.04E+20	PDK4	0.19844358	UBE2C	0.939126194	FBN1	120.6333333
C3	32	PTTG1	57.352	FOXM1	2.04E+20	BUB1B	0.19844358	UBE2S	0.92731216	TIMP2	120.0833333
SPARC	32	ITGB1	57.334	C3	2.04E+20	CDC20	0.19844358	CDC20	0.916924674	APOE	119.9333333
RRM2	32	RRM2	56.74	TRIP13	2.04E+20	CCNA2	0.19844358	FOXM1	0.909318199	HSPB1	119.6166667
COL1A2	31	TYMS	56.212	SMC2	2.04E+20	UBE2C	0.19844358	TGFBI	0.892733263	RRM2	119.5

Supplementary table 2. Top 20 in network ranked by BottleNeck , Betweenness ,Stress,MNC , Radiality methods

BottleNeck method		Betweenness method		Stress method		MNC method		Radiality method	
Name	Score	Name	Score	Name	Score	Name	Score	Name	Score
FN1	32	FN1	12138.33271	FN1	76274	FN1	71	FN1	6.019638243
HIF1A	21	MMP9	6298.562836	MMP9	44738	CDK1	47	MMP9	5.879556164
AURKA	19	CDK1	3142.809591	CDK1	21644	MMP9	43	CXCL8	5.743365253
ILF2	17	AURKA	2820.712022	CCNB1	20602	CCNB1	39	CDH2	5.739474084
HSPB1	13	EZH2	2646.943474	EZH2	20458	COL1A1	39	ITGB1	5.727800578
COL1A1	11	FLNA	2628.306034	AURKA	19444	AURKB	38	AURKA	5.712235902
MMP9	10	CCNB1	2608.408457	HIF1A	19260	ITGB1	38	AURKB	5.708344733
THBS1	9	VCAM1	2428.576176	THBS1	19200	CCNA2	36	CCNB1	5.688888889
CXCL8	9	ITGB1	2402.829229	ITGB1	18776	AURKA	36	THBS1	5.68499772
CP	9	ILF2	2392.908777	VCAM1	17330	BUB1B	35	HIF1A	5.673324213
RRM2	8	HIF1A	2300.835192	CDH2	17040	CDC20	34	HSPB1	5.665541876
CCNB1	7	THBS1	2238.586872	FLNA	16830	TOP2A	34	TIMP1	5.622739018
VCAM1	7	APOE	2184.504243	CXCL8	16552	UBE2C	32	LMNA	5.618847849
UBE2C	7	CXCL8	2124.922684	AURKB	16244	SPARC	32	CDK1	5.611065511
APOE	7	RAP1B	2082.5831	FOXM1	14676	CCNB2	31	TIMP2	5.611065511
C3	6	CDH2	2080.10985	ILF2	14520	COL1A2	31	FOXM1	5.607174343
ITGB1	6	UBE2C	2031.508565	UBE2C	14084	CDH2	31	FLNA	5.599392005
EZH2	6	HSPB1	1991.695704	APOE	13788	FBN1	31	CCNA2	5.587718498
RHOQ	6	ANLN	1982.692191	HSPB1	12756	MAD2L1	30	COL1A1	5.587718498
FLNA	6	CP	1905.281172	COL1A1	12632	PTTG1	30	LAMB1	5.564371485

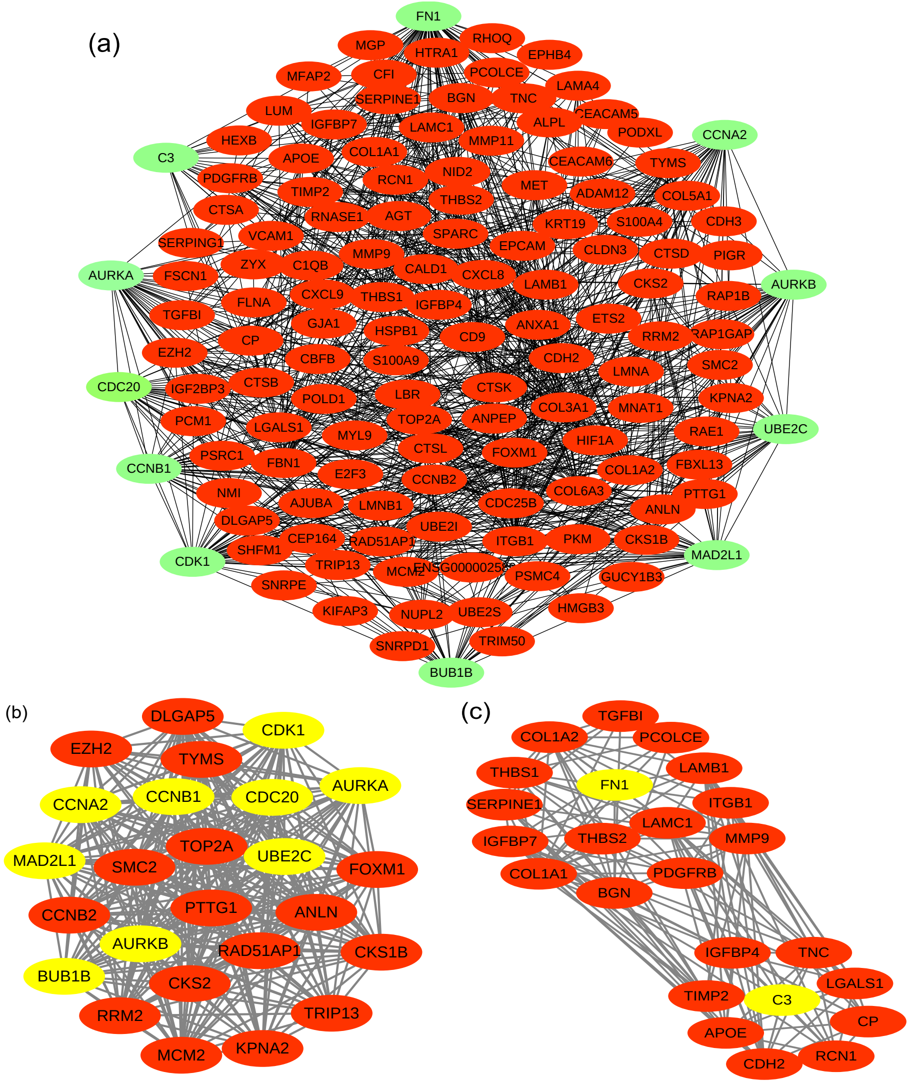

Supplementary Figure 1. PPI network and clustering module related to hub genes. (a) Clustering module regulated by 11 hub genes. Green nodes indicate hub genes and red nodes represent DEGs in GC. (b)-(c) The highest score clustering modules generated by MCODE, yellow color indicates hub genes, red nodes represent DEGs.(The figures were created by Cytohubba and MCODE plug-in in Cytoscape (https://cytoscape.org/))
